# Mild cognitive impairment is associated with poor gait performance in patients with Parkinson’s disease

**DOI:** 10.3389/fnagi.2022.1003595

**Published:** 2022-10-04

**Authors:** Sung Hoon Kang, Jinhee Kim, Jungyeun Lee, Seong-Beom Koh

**Affiliations:** Department of Neurology, Korea University Guro Hospital, Korea University College of Medicine, Seoul, South Korea

**Keywords:** Parkinson’s disease, cognitive impairment, gait disturbance, attention, frontal executive function

## Abstract

Cognitive impairment may be commonly accompanied by gait disturbance in patients with Parkinson’s disease (PD). However, it is still controversial whether gait disturbance is associated with mild cognitive impairment (MCI) and which cognitive function has a more important effect on specific gait parameter. Our objective was to investigate the association of gait parameters with MCI and the correlation between performance on comprehensive neuropsychological tests and gait parameters in PD patients. We enrolled 257 patients with *de novo* PD (111 PD-normal cognition and 146 PD-MCI). All patients underwent comprehensive neuropsychological tests and gait evaluation using the GAITRite system. We used logistic regression analysis and partial correlation to identify the association between gait parameters and MCI and correlations between neuropsychological performance and gait parameters. Gait velocity (odds ratio [OR] = 0.98, 95% confidence interval [CI] = 0.97−0.99) and stride length (OR = 0.98; 95% CI = 0.97−0.99) were associated with MCI in patients with PD. Specifically, gait velocity, stride length, and double support ratio were only associated with attention and frontal-executive function performance in patients with PD. Our findings provide insight into the relationship between gait disturbance and MCI in patients with PD. Furthermore, the evaluation of gait disturbance is necessary for PD patients with cognitive impairment.

## Introduction

Parkinson’s disease (PD) is the second most common neurodegenerative disease and is characterized by progressive motor deficits, including bradykinesia, resting tremor, rigidity, and gait disturbance.

Although gait disturbance is a dopa-responsive symptom at early stage of PD, the response attenuates as the disease progresses (Morris et al., 1994), which in turn leads to debilitating features including freezing of gait and/or falls in patients with advanced PD (Lord et al., 2014). Recently, it has been recognized that non-motor symptoms including cognitive decline commonly occur in PD. The prevalence of mild cognitive impairment (MCI) in patients with PD have found to range from approximately 40–60% at the time of diagnosis (Baiano et al., 2020).

Patients with PD-mild cognitive impairment (PD-MCI) are at a higher risk of poor prognosis including gait disturbance (Fereshtehnejad et al., 2015). Furthermore, cognitive control is needed to compensate for impaired gait function in patients with PD. Given that these findings, the association between cognitive impairment and gait disturbance has become emerged. Specifically, previous studies have found that specific gait parameters are associated with cognition in patients with PD (Rochester et al., 2004, 2005; Wild et al., 2013; Yogev-Seligmann et al., 2013). However, because other studies have shown null results (Rochester et al., 2008; Amboni et al., 2012; Lord et al., 2014), it is still controversial whether gait disturbance may be associated with the presence of MCI and which cognitive function has a more important effect on specific gait parameters in patients with PD. Previous studies had relatively small sample sizes and focused only on global cognitive or executive function; therefore, they did not cover other cognitive domains.

Motor phenotype in patients with PD can be classified into akinetic rigid PD (AR) and tremor-dominant PD (TD). Patients with AR suffer from bradykinesia, rigidity and/or axial symptoms, while the TD is characterized by a tremor. Recently, there is a growing body of evidence that early stage of patients with AR are more likely to have poor prognosis including faster cognitive decline, higher risk of developing dementia, and severe gait disturbance than those with TD (Fereshtehnejad et al., 2015; Wojtala et al., 2019; Yang et al., 2021). However, despite of the differences in severity of cognitive impairment and gait disturbance between the motor phenotype, previous studies did not consider whether gait parameters were associated with specific cognitive functions in relation to motor phenotype.

Therefore, the first objective of our study was to check the differences the relationship between gait parameters and MCI in relation to motor phenotype. The second objective of our study was to investigate the association of gait parameters and the presence of fall or freezing of gait with mild cognitive impairment (MCI) in patients with PD. The second objective was to explore the correlation between performance in comprehensive neuropsychological tests and gait parameters in patients with PD. Given that patients with PD have impaired gait function and then cognitive compensation is necessary to keep gait performance, we hypothesized that MCI was associated with poor gait performance in patients with PD.

## Materials and methods

### Study participants

We recruited 257 patients with *de novo* PD (167 with PIGD and 90 with TDP) at the Movement Disorder Clinic of Korea University, Guro Hospital in Seoul, Korea, from January 2018 to December 2020. All patients underwent comprehensive movement disorder evaluations, including the Unified Parkinson’s Disease Rating Scale (UPDRS), Non-Motor Symptoms Scale (Koh et al., 2012), brain magnetic resonance imaging, standardized neuropsychological tests (Seoul Neuropsychological Screening Battery 2nd edition) (Kang et al., 2019, 2021b), and comprehensive gait evaluation using the GAITRite system (CIR System Inc., USA) before the initiation of PD medication. The time interval between the comprehensive neuropsychological test and gait evaluation was less than 1 month. We excluded patients who exhibited any of the following conditions: (1) Parkinsonism due to offending drugs; (2) history of PD treatment in other hospitals; (3) dementia on a comprehensive neuropsychological test; (4) severe white matter hyperintensity (WMH) defined as deep WMH ≥ 25 mm and periventricular WMH ≥ 10 mm on fluid-attenuated inversion recovery image; (5) territorial infarction, lobar hemorrhage, brain tumor, and hydrocephalus or other structural lesions; and (6) history of psychiatric illness, including major depressive disorder, bipolar disorder, and schizophrenia.

Patients with PD were dichotomized as TD or AR using a modified ratio based on UPDRS III (Schiess et al., 2000; Kang et al., 2016). Specifically, the tremor/akinetic-rigidity ratio was calculated from the patient’s mean tremor and akinetic-rigidity scores. Tremor was assessed using a nine-item scale that included a history of left or right tremor (two items), rest tremors of the face/lips/chin and each limb (five items), and postural tremor of the right and left upper extremities (two items). Akinetic-rigidity was assessed using a 12-item scale that included passive range of motion rigidity of the neck and each extremity (five items), rapid opening/closing of the hands (one item), finger tapping (one item), rising from a chair (one item), posture and postural instability (two items), gait (one item), and body bradykinesia (one item). Each item was rated 0–4, with 0 representing the absence of symptoms/normal activity and 4 representing the significant presence of the symptom. Mean tremor and akinetic-rigidity scores were calculated, and then the ratio (tremor/akinetic-rigidity score) was determined. Patients with a ratio >1.0 were defined as TD, while those with a ratio ≤1.0 were defined as AR.

All participants with PD were patients with PD-normal cognition (PD-NC) and those with PD-MCI. All patients with PD-NC met the following criteria: (1) diagnosis of PD based on the UK Brain Bank criteria (Hughes et al., 1992); (2) no objective cognitive impairment or objective cognitive impairment in only one neuropsychological test; (3) no medical history likely to affect cognitive function based on Christensen’s health screening criteria (Christensen et al., 1991); and (4) no significant impairment in activities of daily living. All patients with PD-MCI met the MDS level II criteria for PD-MCI: objective cognitive impairment below –1.0 SD in at least two neuropsychological tests, in which two tests were impaired in one cognitive domain or one test was impaired in two different cognitive domains (Litvan et al., 2012).

This study was approved by the Institutional Review Board of Korea University, Guro Hospital. Written informed consent was obtained from all patients.

### Assessment of gait disturbance related symptoms and gait parameters

Patients reported symptoms of falling and freezing of gait during interviews. Falling was assessed using the question, “did you have a history of falls during the past year?” (Pickering et al., 2007). The freezing of gait was assessed using the FOG questionnaire, and an FOG questionnaire item score ≥ 1 was defined as having freezing of gait (Giladi et al., 2000).

Patients underwent a comprehensive gait evaluation regarding spatial and temporal parameters of gait dynamics using the GAITRite system with a 4.6-m-long walkway (Kang et al., 2021a). Average spatiotemporal parameters, such as velocity, stride length, cadence, step length coefficient of variation (CV), and double support ratio, were measured during 10-times forward walking.

### Assessment of cognitive function

All patients underwent neuropsychological tests using the Seoul Neuropsychological Screening Battery 2nd edition (Kang et al., 2019). We opted to use 10 cognitive measurements of representative and important neuropsychological tests to evaluate cognitive function in five cognitive domains as follows: (1) attention, the Digit Span Test backward and Stroop Test (word reading); (2) language, the Korean version of the Boston Naming Test (K-BNT) and animal component of the Controlled Oral Word Association Test (COWAT); (3) visuospatial function, the Rey–Osterrieth Complex Figure Test (RCFT) copying Test and Clock Drawing Test; (4) memory, the Seoul Verbal Learning Test (SVLT) delayed recall (verbal memory) and RCFT delayed recall (visual memory); and (5) frontal executive function, the phonemic component of the COWAT and the Stroop Test (color reading). Continuous numerical values were converted to z-scores using the standardized norms for age and education presented in the SNSB-II, and z-scores were used in the analysis.

### Statistical analyses

We used the independent *t*-test and chi-square test to compare the demographic and clinical characteristics between the PD-NC and PD-MCI groups. To assess the interaction effect of cognitive status (PD-NC or PD-MCI) and motor phenotype (AR or TD) on gait disturbance, we performed a two-way ANOVA with interaction test on gait parameters (velocity, stride length, cadence, step length CV, and double support ratio). To determine the association of gait-related symptoms (easy falling, freezing of gait, and gait ignition failure) and gait parameters (velocity, stride length, cadence, step length CV, and double support ratio) with MCI, we used logistic regression, including the presence of gait-related symptoms (easy falling, freezing of gait, and gait ignition failure) or gait parameters (velocity, stride length, cadence, step length CV, and double support ratio) as separate independent variables after controlling for age, sex, disease duration and motor phenotype. Finally, we used partial correlations after controlling for age, sex, disease duration, and motor phenotype to explore the correlation between gait parameters and specific neuropsychological performance.

All reported *p*-values were two-sided, and the significance level was set at 0.05. All analyses were performed using R version 3.6.1 (Institute for Statistics and Mathematics, Vienna, Austria).^1^

## Results

### Clinical characteristics of the study participants

Among the 257 patients with PD, 111 were PD-NC and 146 were PD-MCI. Patients with PD-MCI were older than those with those with PD-NC (*p* = 0.036). The proportion of females (*p* = 0.056), disease duration (*p* = 0.611), ratio of easy falling (*p* = 0.215) and gait ignition failure (*p* = 0.288), cadence (*p* = 0.161), step length variability (*p* = 0.812), and double support ratio (*p* = 0.051) did not differ between the PD-NC and PD-MCI groups (Table 1). Patients with PD-MCI were more likely to experience freezing of gait (*p* = 0.028) and had slower velocity (*p* = 0.014), and shorter stride length (*p* = 0.021) than those with PD-NC (Table 1).

**TABLE 1:** Demographic variables and gait profiles of study participants.

	PD-NC (*n* = 111)	PD-MCI (*n* = 146)	*P-value*
**Motor phenotype**			
AR/TD	40 (36.0%)/71 (64.0%)	50 (34.2%)/96 (65.8%)	0.766
**Demographics**			
Age (years)	69.8 ± 7.3	71.9 ± 8.0	0.036
Sex, females	71 (64.0%)	76 (52.1%)	0.056
Education (years)	8.4 ± 4.7	8.7 ± 4.9	0.677
Disease duration	25.8 ± 27.1	27.6 ± 27.	0.611
Hypertension	51 (45.9%)	72 (49.3%)	0.592
Diabetes	21 (18.9%)	37 (25.3%)	0.222
Easy falling	6 (5.4%)	14 (9.6%)	0.215
Freezing of gait	7 (6.3%)	22 (15.1%)	0.028
Gait ignition failure	6 (5.4%)	13 (8.9%)	0.288
**Gait parameters**			
Velocity (m/s)	84.5 ± 20.5	77.7 ± 23.1	0.014
Stride length (m)	94.8 ± 20.0	88.3 ± 23.8	0.021
Cadence	107.8 ± 14.6	105.6 ± 10.8	0.161
Step length CV	2.6 ± 2.6	2.5 ± 2.2	0.812
Double support (%)	30.4 ± 5.4	32.0 ± 7.2	0.051

Values are presented as the mean ± standard deviation. PD-NC, Parkinson’s disease-normal cognition; PD-MCI, Parkinson’s disease-mild cognitive impairment; AR, akinetic rigid-type PD; TD, tremor-dominant-type PD; CV, coefficient of variation.

The interaction effects of cognitive status (PD-NC or PD-MCI) and motor phenotype (AR or TD) on gait parameters were not significant (Table 2).

**TABLE 2:** Differences in gait parameters in relation to cognitive status and motor phenotype.

Variables	Interaction effect (cognitive status × motor phenotype)	Main effect 1 (cognitive status)	Main effect 2 (motor phenotype)
	*F*-value/*p*-value*	*F*-value/*p*-value	*F*-value/*p*-value
Velocity	2.08/0.151	6.65/0.011	23.53/ < 0.001
Stride length	2.66/0.104	6.05/0.015	30.42/ < 0.001
Cadence	0.09/0.765	1.96/0.162	0.01/0.935
Step length CV	0.05/0.821	0.06/0.811	5.45/0.020
Double support	1.88/0.172	3.49/0.063	14.79/ < 0.001

*p for interaction effect was obtained using the two-way ANOVA with interaction test (diagnosis × motor phenotype) on gait parameters. CV, coefficient of variation.

### Association of gait related symptoms or gait parameters with Parkinson’s disease-mild cognitive impairment

Freezing of gait (odds ratio [OR] = 2.84; 95% confidence interval [CI] = 1.08−7.43), slower gait velocity (OR = 0.98; 95% CI = 0.97−0.99), and shorter stride length (OR = 0.98; 95% CI = 0.97−0.99) were associated with PD-MCI (Table 3).

**TABLE 3:** Odds ratio for PD-MCI in patients with PD.

	PD-MCI	
	OR* (95% CI)	*p*
**Symptoms**		
Easy falling	1.68 (0.60–4.72)	0.322
Freezing of gait	2.84 (1.08–7.43)	0.034
Gait ignition failure	1.71 (0.59–4.97)	0.328
**Gait parameters**		
Velocity	0.98 (0.97–0.99)	0.022
Stride length	0.98 (0.97–0.99)	0.021
Cadence	0.99 (0.97–1.01)	0.273
Step length CV	0.98 (0.88–1.09)	0.756
Double support ratio	1.04 (1.00–1.09)	0.070

MCI, mild cognitive impairment; PD, Parkinson’s disease; OR, odds ratio; CI, confidence interval; CV, coefficient of variation. *Adjusted OR for MCI was obtained using logistic regression analyses with each gait-related symptom or gait parameter as a single independent predictor, after controlling for age, sex disease duration, and motor phenotype.

### Relationship between gait parameters and performance in neuropsychological tests

**FIGURE 1:**
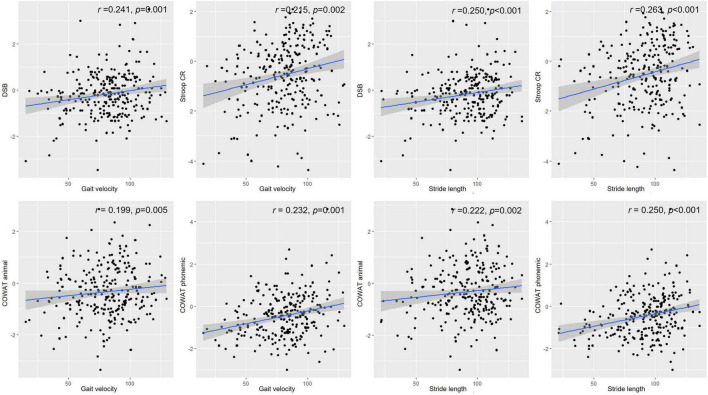
Correlations between gait parameters and neuropsychological performance. Values depicted in the scatter plot represent gait velocity or stride length on the X-axis and score in the Digit Span Test backward, Stroop color reading, animal component of COWAT, or phonemic component of COWAT on the Y-axis. DSB, Digit Span Test backward; Stroop CR, Stroop color reading; COWAT animal, animal component of COWAT; COWAT phonemic, phonemic component of COWAT.

Table 4 shows the correlation between gait parameters and performance on neuropsychological tests. Gait velocity was positively correlated with performance in the Digit Span Test backward (*r* = 0.241, *p* = 0.001), semantic (*r* = 0.199, *p* = 0.005), and phonemic component of the COWAT (*r* = 0.232, *p* = 0.001), and the color reading portion of the Stroop Test (*r* = 0.215, *p* = 0.002) in patients with PD (Figure 1). Stride length was also positively correlated with performance in the Digit Span Test backward (*r* = 0.250, *p* < 0.001), RCFT copying Test (*r* = 0.156, *p* = 0.029), semantic (*r* = 0.222, *p* = 0.002), phonemic component of the COWAT (*r* = 0.250, *p* < 0.001), and color reading portion of the Stroop Test (*r* = 0.263, *p* < 0.001) in patients with PD (Figure 1). The double support ratio was negatively correlated with the performance in the Digit Span Test backward (*r* = –0.195, *p* = 0.006), semantic component of the COWAT (*r* = –0.159, *p* = 0.026), phonemic component of the COWAT (*r* = –0.183, *p* = 0.010), and color reading portion of the Stroop Test (*r* = –0.201, *p* = 0.005) in patients with PD.

**TABLE 4:** Correlation between gait parameters and neuropsychological tests.

	Velocity	Stride length	Cadence	Step length CV	Double support ratio
DST backward	0.241**	0.250***	0.062	–0.105	–0.195*
BNT	0.042	0.101	–0.099	–0.036	–0.051
RCFT copy	0.094	0.156*	–0.082	–0.100	–0.031
SVLT dr	0.039	0.046	–0.017	–0.066	–0.018
RCFT dr	0.065	0.090	–0.049	–0.056	–0.034
COWAT animal	0.199*	0.222**	0.001	–0.064	–0.159*
COWAT phonemic	0.232**	0.250***	0.015	–0.095	–0.183*
Stroop CR	0.215**	0.263***	–0.012	–0.134	–0.201*

CV, coefficient of variation; DST, Digit Span Test; BNT, Boston Naming Test; RCFT, Rey–Osterrieth Complex Figure Test; SVLT, Seoul Verbal Learning Test; COWAT, Controlled Oral Word Association Test; CR, color reading. *p-value < 0.05, **p-value < 0.005, ***p-value < 0.001.

## Discussion

In this large cohort study, we investigated the association between gait parameters and cognitive function in patients with *de novo* PD. The main findings of this study were as follows. First, gait velocity or stride length were associated with PD-MCI. Second, poor cognitive performances in attention and frontal-executive function were associated with slower gait velocity, shorter stride length, and higher double support ratio in patients with PD. Taken together, our findings provide insight into the relationship between gait disturbance and the presence of MCI in patients with PD.

Patients with AR have a more widespread PD pathology burden (Nutt, 2016) and worse gait performance than those with TD, and therefore cognitive compensation is more needed in AR motor phenotype, we expected that the relationship between gait disturbance and MCI was more stronger in patients with AR than TD. However, in contrary to our expectations, we found that the relationship of gait disturbance with MCI was not different between patients with AR and TD.

Our first major finding was that slower gait velocity or shorter stride length was associated with MCI in patients with PD. These results are consistent with that of previous findings, which demonstrated that patients with PD-MCI showed slower velocity and shorter stride length than those with PD-NC (Morris et al., 1996; Amboni et al., 2012). Shared neural substrate degeneration and neurotransmitter deficits may explain our findings. Specifically, fronto-striatal neurodegeneration causes gait disturbance and subcortical-type cognitive impairment (Pillon et al., 1993) in patients with PD. Previous studies have also reported that hypometabolism in the prefrontal cortex was observed in the early stages of PD (Chu et al., 2019), which correlates with gait disturbance and cognitive impairment (Stuart et al., 2020). Given that there is growing evidence that the cholinergic system may be a pivotal contributor not only to cognitive performance but also to gait function in PD (Bohnen et al., 2011; Müller and Bohnen, 2013), cholinergic denervation in the basal forebrain and pedunculopontine may be another reason for the association between cognition and gait. Recent studies have supported that cholinergic deficits in the basal forebrain and mesencephalic locomotor area are responsible for cognitive impairment and gait disturbance including falls and freezing of gait (Bohnen et al., 2009, 2014).

Our second major finding was that poor cognitive performances in attention and frontal-executive function were associated with slower gait velocity, shorter stride length, and higher double support ratio only in patients with PD. Our results are consistent with that of previous studies showing that gait-related cognitive domains include attention and frontal-executive function, which are mainly correlated with velocity and stride length (Lord et al., 2010). This relationship may be explained by the facts that cognitive compensation to maintain the gait function is closely related to attention and frontal-executive function. Gait, an automatic motor skill to control movement in healthy individuals, is impaired in patients with PD, leading to an increased demand for attention and frontal-executive function (Yogev et al., 2005). In fact, patients with PD showed higher prefrontal activity during walking to compensate for gait control than healthy individuals (Stuart et al., 2019; Stuart and Mancini, 2020). Given that the prefrontal cortex is a well-known neural substrate for attention and frontal-executive function (Kang et al., 2019, 2021b), cognitive demand on attention and frontal-executive function may increase during walking in patients with PD.

Our study had several limitations that should be addressed. First, because this study had a cross-sectional design, a causal relationship between gait disturbance and cognitive impairment could not be concluded. Second, we did not address the effects of dual tasks on gait. Dual tasks are considered a sensitive condition to detect underlying gait disturbance and cognitive impairment in patients with PD because dual tasks aggravate gait performance, and the magnitude of worsening may be associated with underlying cognitive function. Finally, given that 59 (23.0%) out of 257 patients with PD had less than 1-year follow-up period, we could not completely rule out the possibility that a few patients diagnosed with PD at first would progress to multiple system atrophy or progressive supranuclear gaze palsy. However, we excluded the participants who had red flag sign including supranuclear gaze palsy, cerebellar sign, Babinski sign with hyperreflexia, stridor and atrophy of putamen, superior cerebellar peduncle, middle cerebellar peduncle, midbrain, pons or cerebellum on MRI at baseline evaluation to increase the diagnostic accuracy of *de novo* PD. However, our study is noteworthy because we comprehensively reported an association between cognitive impairment and gait disturbance in a large number of patients with *de novo* PD. Our findings suggest that the evaluation of gait disturbance is necessary for PD patients with cognitive impairment, while further longitudinal studies are needed to understand the complex mechanism of our findings.

## Data availability statement

The raw data supporting the conclusions of this article will be made available by the authors, without undue reservation.

## Ethics statement

The study protocol was reviewed and approved by the Institutional Review Board of Korea University Guro Hospital. The patients/participants provided their written informed consent to participate in this study.

## Author contributions

SK analyzed and interpreted the data and drafted the manuscript for intellectual content. JK and JL played major roles in data acquisition. S-BK acquired the data, designed and conceptualized the study, and revised the manuscript for intellectual content. All authors contributed to the article and approved the submitted version.

## Footnotes

1
www.R-project.org

## Abbreviations

AR: akinetic rigid PD
COWAT: controlled oral word association test
CV: coefficient of variation
K-BNT: Korean version of the Boston naming test
MCI: mild cognitive impairment
PD-NC: Parkinson’s disease-normal cognition
RCFT: Ray Osterrieth complex figure test
SVLT: Seoul Verbal Learning Test
TD: tremor-dominant PD
UPDRS: Unified Parkinson’s Disease Rating Scale
WMH: white matter hyperintensity.
